# Evaluation of the Doxycycline Release from AH26 Sealer-Doxycycline Combination: An ex vivo Study

**Published:** 2011-11-15

**Authors:** Kazem Ashofteh Yazdi, Noushin Shokouhinejad, Esmaeil Moazeni, Sina Mirzayi Rad

**Affiliations:** 1. Department of Endodontics, Dental Research Center/Dental School, Tehran University of Medical Sciences, Tehran, Iran.; 2. Department of Endodontics, Dental Research Center/ Dental School, Tehran University of Medical Sciences, and Iranian Center for Endodontic Research, Tehran, Iran.; 3. Department of pharmaceutics, Pharmacy School, Tehran University of Medical Sciences, Tehran, Iran.; 4. Department of Endodontics, Dental School, Babol University of Medical Sciences, Babol, Iran.

**Keywords:** AH26 Sealer, Doxycycline, Root Canal

## Abstract

**INTRODUCTION:**

The purpose of this ex vivo study was to determine the releasing characteristics and doxycycline dentinal diffusion of AH26 sealer-doxycycline combination from apical 3mm of tooth root and apical foramen.

**MATERIALS AND METHODS:**

One-hundred and two recently extracted single-rooted human teeth were decoronated and prepared with #3 and #4 Gates-Glidden drills and rotary Mtwo files. Smear layer was removed; all surfaces except for apical 3mm of each root were sealed with two coats of nail polish. To quantify the release and diffusion of the doxycycline at different time intervals (30 min, 48 and 72 h) after root canal obturation, the samples were randomly divided into three groups (n=30; 0.5 h, 48 h, 72 h). To evaluate the release of doxycycline from AH26 sealer-doxycycline combination at six concentrations of antibiotic including 0.5%, 1%, 2%, 5%, 10% and 20%; each experimental group was divided into six equal subgroups (n=5). Root canals were filled with gutta-percha and AH26-doxycycline combinations and then were placed in vials containing 1.25mL of phosphate buffer saline solution (PBS). After 30 min, 48 and 72 h, the amount of doxycycline released from specimens into PBS were determined by measuring the absorbance values using UV spectrophotometry at λ_max_=350 nm. Data were analyzed using two-way ANOVA.

**RESULTS:**

The findings of this study revealed that AH26 sealer-doxycycline combination released variable measures of antibiotic at each time interval and in the various concentrations. At 30 min, no statistically significant differences were obtained between the results of subgroups, but at 48 and 72 h these differences were significant (P<0.001). The results also showed that differences between 0.5 h, 48 h and 72 h were significant within subgroups (P<0.01).

**CONCLUSION:**

Under the conditions of this ex vivo study, doxycycline can be released from AH26 sealer-antibiotic combination through 3mm of apical root and apical foramen at 30 min, 48 and 72 h after mixing the sealer with doxycycline at concentrations of 0.5% up to 20%.

## INTRODUCTION

Bacteria and their by-products are considered to be the most common causes of pulpal necrosis and periapical lesions. In the majority of teeth requiring root canal treatment, the goal is either prevention or treatment of apical periodontitis, or more precisely, prevention or elimination of a microbial infection in the root canal system [1].

Within the last few years, antibiotics have been used for patients in dentistry systemically and topically. Chronic periradicular lesions associa-ted with necrotic pulp do not have adequate blood supply. So when systemic antibiotic treatment is being used, the concentration of antibiotics that reach root canal system is negligible and not beneficial [2]. Systemically administered antibiotics also can have some complications such as toxicity, allergic reactions and development of resistant strains of micro-organisms [3]. It has been reported that the main advantage of local antibiotics compared to systemic use is absence of systemic complica-tions; therefore, substantially higher concentra-tions of antibiotics can be used [4].

Also, the filling of a prepared root canal with an antimicrobial medicament allows the antibiotic to diffuse through the dentin, the canal irregularities and also into the periapical tissues. This then exposes the bacteria present in these areas to the medicaments and can enhance repair of a periapical lesion by reducing the number of active bacteria and/or other effects of medicaments such as anticollagenase and anti-resorptive activity of several antibiotics (e.g. tetracycline) [5]. Tetracyclines’, including tetracycline HCl, minocycline, demeclocycline and doxycycline, are a group of broad spectrum antibiotics that are effective against a wide range of microorganisms. Tetracyclines also have many additional properties, such as the inhibition of mammalian collagenases so that prevent tissue breakdown, and the inhibition of clastic cells so they provide anti-resorption result. Inflammatory diseases such as periodontitis include an excess of tissue collagenases, which may be blocked by tetracyclines, thus leading to enhanced formation of collagen and bone [5].

AH26 is an epoxy resin root canal sealer that initially was developed as a single-filler material. Because of its positive handling characteristics, it has been extensively used as a sealer. It has favorable flow, adaptation to dentinal walls, and working time [6]. Like most sealers, AH26 is very toxic when freshly prepared. However, its toxicity declines rapidly during setting reaction and has the lowest toxicity among endodontic sealers after 24 h [7].

There are few studies that have assessed the incorporation of antibiotics to endodontic sealers; they have evaluated the antimicrobial effects of antibiotic-sealer combinations against Enterococcus faecalis and physicomechanical properties of AH26/antibiotic combination [8][9][10].

The purpose of this ex vivo study was to determine the release of doxycycline from AH26 sealer-doxycycline combination through apical 3mm of root and apical foramen.

## MATERIALS AND METHODS

One hundred and two recently extracted straight single-rooted human teeth with approximately same dimensions were selected and stored in saline solution. These teeth had the following characteristics: single canal, no fractures or caries, root resorption and open apices. The teeth were decoronated using diamond disks (D&Z, Berlin, Germany) to obtain a standardized root length of 14mm. The root canal of each tooth was explored using a size 10 or 15 K-file (Dentsply, Maillefer ,Tulsa, OK, USA) until the apical foramen was reached and the tip of the file was visible. The actual canal length was determined and WL was established by subtracting 1mm from this measurement. After coronal flaring with #3 and #4 Gates-Glidden drills (Dentsply, Maillefer, Tulsa, OK, USA), instrumentation was completed with Mtwo rotary files (VDW, Munich, Germany) up to file size 40 in a step-back technique. A K-file size 10 was used to ensure apical patency between each file. Canals were irrigated with 5.25% NaOCl throughout the instrumentation. Then, a K-file size 30 was introduced into the root canal until the file tip was visible (standardization of apical foramen) and then canals were irrigated with 1mL of 17% EDTA for 1min and 1mL of 5.25% NaOCl to remove the smear layer. Finally, root canals were dried with paper points. All surfaces except for the apical 3mm of root were sealed with two coats of nail polish.

In order to quantify the release and diffusion of the doxycycline at different times (30 min, 48 and 72 h) after root canal obturation, the samples were randomly divided into three groups of 30 specimens (0.5 h, 48 h, 72 h) For evaluating the release of doxycycline from AH26 sealer-doxycycline combination at six concentrations of 0.5% (DX0.5), 1% (DX1), 2% (DX2), 5% (DX5), 10% (DX10), 20% (DX20) of antibiotic, each experimental group was divided into six equal subgroups (n=5). At each time period, three specimens were obturated with AH26 sealer (Dentsply, DeTrey, Konstanz, Germany) and gutta-percha (control group 1) and one tooth was not obturated (control group 2).Doxycycline (Iran Daru, Tehran, Iran) was added separately to the powder of AH26 sealer (Dentsply, DeTrey, Konstanz, Germany) in each concentration and mixed with liquid of sealer according to manufacturers’ specifications. Then the root canals were filled with gutta-percha and AH26-doxycycline combinations; gutta-percha was removed up to 2mm below the orifices and the spaces were sealed with glass ionomer filling material (Fuji IX, GC Co., Tokyo, Japan) and were then covered with dental adhesive wax.

After this stage, specimens were placed in 1.5mL eppendorf vials containing 1.25mL of phosphate buffered saline (PBS) and then roots were stored vertically at room temperature.

After 30 min, 48 and 72 h the amounts of doxycycline released from specimens to PBS were determined by measuring the absorbance values using UV spectrophotometry at λmax=350nm. The apparatus was calibrated at zero absorbance by using PBS as blank. The details for these steps are exampled below:

### Graphing the standard curve of doxycycline

A standard curve is a graph relating a measured quantity (spectrophotometry, for example) to concentration of the substance of interest in "known" samples.

### Preparation of standard stock solution

A standard stock solution of doxycycline was prepared by weighing 10mg of the drug and dissolving this in 100mL distilled water. The stock solution was diluted in the concentration range of 1-50μg/mL with distilled water to obtain the concentration ranges required.

### Determination of λ_max_

To determine the λ_max_ of doxycycline (wavelength that maximum absorption occurred), two concentration of prepared solutions was assessed by UV spectrophotometry. The value of λ_max_ for doxycycline was determined at 350nm. The amount of doxycycline dissolved in each sampled solution was determined by UV spectrophotometry (JASCO Co. Ltd., Tokyo, Japan) at λ_max_.

The standard curve was drawn by plotting absorbance (on the Y axis) vs. concentration (on the X axis). Such a curve can be used to determine concentrations of the doxycycline in "unknown" samples. The points on the calibration curve should yield a straight line (regression line). To validate the standard curve, this procedure was repeated three times on the same day and another two times on two different days.

To eliminate or reduce the interfering factors with absorbance, the final absorbance was calculated from these data after the control group absorbance was subtracted. The absorbance of the doxycycline was then used with the slope and intercept from the calibration curve to calculate the concentration of the doxycycline (μg/mL). These concentrations (RDX) were also reported as μg per 1.25mL.

All of this procedure was repeated three times and the mean values of doxycycline concentration were calculated for each experimental group.

Two-way analysis of variance (ANOVA) was used in testing the effect of time, antibiotic concentration, and their interactions on doxycycline release. The level of significance was set at P<0.05.

## RESULTS

**Figure 1:**
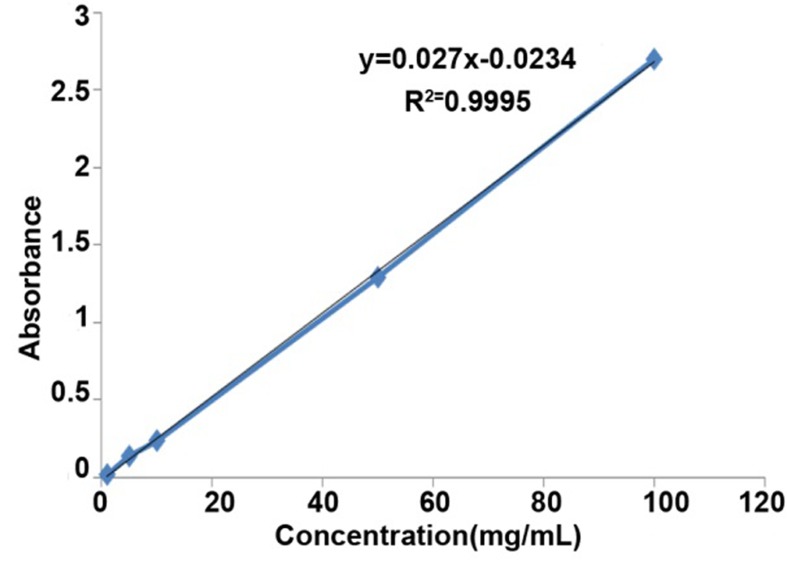
Standard curve of doxycycline

Figure 1 shows the standard curve of doxycycline that has been drawn by plotting absorbance (Y axis) vs. concentration (X axis). The linearity of the calibration curve for doxycycline over the concentration range 1-100μg/mL demonstrated a correlation coefficient of 0.9995.

Specimens of control group 1 showed some degrees of UV absorbance at λ_max_=350nm; however, this was not observed in PBS obtained from control group 2.

Table 1 shows the mean values of doxycycline that were released in each experimental group RDX) as μg/mL and μg/1.25mL and their standard deviations.

**Figure 2:**
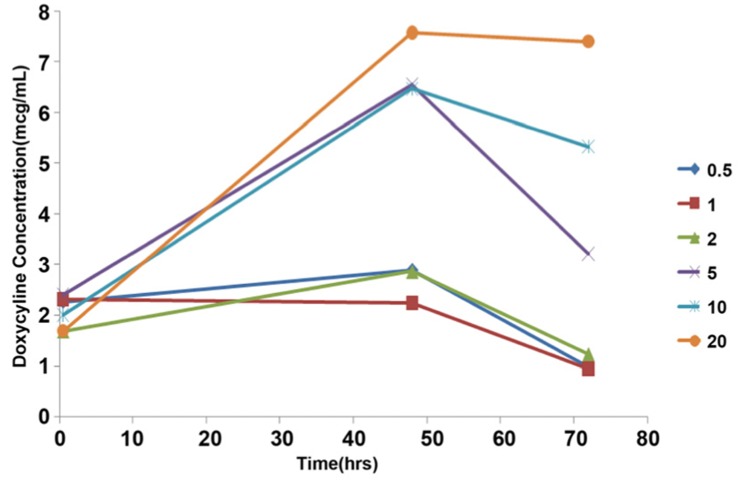
The ex vivo release of doxycycline at different times from AH26-doxycycline combination in six concentration

Figure 2 shows the ex vivo release of doxycycline at different times.

**Table 1:** Mean (±SD) values of RDX (Concentration) in each experimental group

**DX**	**RDX (μg/mL)**			**RDX (μg/1.25mL)**		
	**30 min**	**48 h**	**72 h**	**30 min**	**48 h**	**72 h**
**0.5%**	2.270 (±1.40)	2.899 (±1.64)	0.983 (±1.20)	2.837 (±1.75)	3.624 (±2.05)	1.229 (±1.50)
**1%**	2.312 (±0.82)	2.245 (±1.66)	0.944 (±1.23)	2.890 (±1.02)	2.806 (±2.07)	1.181 (±1.57)
**2%**	1.681 (±0.78)	2.867 (±1.44)	1.233 (±1.29)	2.102 (±0.97)	3.58 (±1.8)	1.542 (±1.61)
**5%**	2.410 (±1.10)	6.554 (±2.17)	3.212 (±1.98)	3.013 (±1.37)	8.192 (±2.71)	4.015 (±2.47)
**10%**	2.008 (±0.88)	6.467 (±2.44)	5.328 (±2.15)	2.510 (±1.1)	8.084 (±3.05)	6.661 (±2.68)
**20%**	1.691 (±1.48)	7.575 (±1.87)	7.402 (±2.96)	2.113 (±1.85)	9.468 (±2.33)	9.252 (±3.7)

### Split on time

At 30 min, no statistically significant differences between the results of subgroups (DX0.5, DX1, DX2, DX5, DX10 and DX20) were obtained (P=0.298) but at 48 and 72 h these differences were significant (P<0.001).

At 48 h, there were no significant differences between results of DX0.5, DX1 and DX2 (P>0.05), also no statistically significant differences were found between the results obtained from DX5, DX10 and DX20 (P>0.05), but differences between results of any of former subgroups with any of latter subgroups were significant (P<0.05).

At 72 h, there were no statistically significant differences among the DX0.5, DX1 and DX2 (P>0.05), but significant differences in RDX were found between any of DX0.5 and DX1 subgroups with any of DX5, DX10 and DX20 subgroups (P<0.05). The results showed no significant difference between the DX2 and DX5 (P>0.05) whereas the results of DX2 with DX10 and DX2 with DX20 had significant differences (P<0.05). No significant difference was seen between results of DX5 and DX10 (P>0.05) but difference between DX5 and DX20 was significant (P<0.05). No statistically significant difference between the results of DX10 and DX20 was obtained (P>0.05).

### Split on DX

The results of this study showed that in any of subgroups i.e. DX0.5 (P=0.002), DX1 (P=0.008), DX2 (P=0.002), DX5, DX10 and DX20 (P<0.001) differences between T0.5, T48 and T72 were significant.

In subgroup DX0.5, the result of T72 was significantly lower than that of T0.5 and T48 (P<0.05). However, there was no significant difference between RDX of T0.5 and T48 (P>0.05).

In subgroup DX1, only the difference T0.5 and T72 was significant (P<0.05).

In subgroups DX2 and DX5, the result of T48 was significantly higher than T0.5 and T72 (P<0.05), whereas difference between T0.5 and T72 was not significant (P>0.05).

In subgroups DX10 and DX20, the result of T0.5 was significantly lower than T48 and T72 (P<0.05), but no significant difference was seen between the results of T48 and T72 (P>0.05).

## DISCUSSION

One of the key goals of endodontic therapy is complete obturation of the root canal system. The success of obturation is directly dependent on the elimination of microorganisms through mechanical cleaning and shaping, supplemented by antibacterial irrigant, adequate filling of the root canal system, and use of antimicrobial medicaments between appointments, if necessary. However, these procedures do not result in complete sterility of the root canal space [11]. Therefore, antimicrobial agents are added to the root canal sealers to improve their antibacterial activity. The antibacterial properties of sealers may also be advantageous in cases of persistent or recurrent infection [12].

AH26 was chosen as the test sealer because of its easy handling characteristics, good flow, good sealing to dentin, sufficient working time [7], prominent antimicrobial activity [13][14] and optimal depth of penetration into dentinal tubules in clinical situations [15].

Antiseptics are very effective antimicrobial agents but they also tend to kill mammalian cells at similar concentrations that kill micro-organisms. This toxicity is time dependent so their use should be limited to short-term contact. Antibiotics are less toxic to mammalian cells at effective concentrations and they are not suitable for short-term use [2].

Tetracyclines (such as doxycycline) are substantive due to their hard tissue binding capability and also have an innate anti-resorptive action. They have the ability to bind to the tooth surface and then be slowly released in active form. Tetracyclines also promote fibroblast and connective tissue attachment, enhancing regeneration of periodontal attachment lost to pathologic process. Tetracyclines inhibit the activity of collagenase and also the function of the osteoclast. Their effects on the osteoclast include diminished acid production, decreased ruffled border area and decreased adhesive properties, all of which inhibit bone resorption [16]. Doxycycline was assessed in current study because of these advantageous properties along with its antimicrobial activity.

In this study, the concentration of the doxycycline released into PBS was evaluated by UV spectrophotometry. In many studies this method has been used successfully to determine doxycycline [17][18][19][20][21][22][23].

In the present study, the doxycycline release was evaluated at 30 min, 48 and 72 h after obturation of root canals. In our pilot study, the setting time of AH26 sealer-doxycycline combination was increased and found to be about 50 h these times (30 min, 48 and 72 h) were selected on the basis of pilot data.

The amount of doxycycline that should be added to sealer was determined according to results of two previous studies [8][9] and our pilot experiment.

Varying degrees of UV absorbance were observed in control group 1. This finding was because of the low specificity of UV spectrophotometry in determination of doxycycline in solutions that two or more interfering components with overlapped absorption bands are available in them [24]. The interfering factor may be formaldehyde that is released from sealer into PBS [25]. In our study, to eliminate or reduce the interfering factors with absorbance, the final absorbance was calculated from data of experimental groups after the control group’s absorbance were subtracted [26].

The result of our study revealed that in 0.5 h, differences between any of subgroups were not significant. The shortest evaluation time after obturation was 30 min and in this time the amount of doxycycline that was available in sealer could not be very drastic variable, but in T48 and T72 there were sufficient time for this variable to make difference between RDX of subgroups.

Our results did not reveal a completely similar pattern of increase in subgroups and this finding might be attributed to differences between specimens from aspects of tooth age, dentinal tubule diameter, number of lateral canal that are present in apical 3mm of root and presence or absence of cementum [22][23].

The results of this study also showed that in any of subgroups, differences between T0.5, T48 and T72 were significant. In any amount of DX, RDX increased from 30 min to 48 h and then decreased. This finding may be attributed to balance between the amount of release and stability of drug in PBS [27]. Furthermore, it is logical that the release of drug decreases after setting of sealer. It should be kept in mind that in current study, specimens were separately placed in PBS and the release of drug was not evaluated in one specimen over time.

Additionally, the hard tissue-binding capability of doxycycline should be considered as a potential factor that can influence the release characteristics of drug. No data are available about the range of time that maximum binding is occurred.

Nevertheless, it is important to note that doxycycline is one of the most stable tetra-cycline group members and a good candidate for slow release formulations [27].

Abbott et al. showed that the rate of demeclo-cycline release from ledermix paste was highest during the first day and declined exponentially with time thereafter [22]. These findings are in agreement with our study.

In many studies, the antimicrobial suscepti-bilities of pathogens isolated from odontogenic and periodontal infections were determined. Slots et al. reported that minimum inhibitory concentrations (MICs) of doxycycline for periodontal pathogens were lower than 6μg/mL [28]. However, Solvi et al. stated that susceptibilities of periodontal pathogens to doxycycline were in the range of 0.1-2μg/mL [29]. In another study, Mosca et al. evaluated the antimicrobial susceptibility of Prevotella spp. And Fusobacterium nucleatum isolated from patients with periodontal disease [30]. MIC90 of doxycycline for F nucleatum, P intermedia, P bucca and P melanogenica were 0.38, 0.094, 0.094 and 4μg/mL, respectively. In a study by Norris and Love, the antimicrobial suscepti-bilities of three Porphyromonas spp to doxycycline were determined 8μg/mL [31]. Carson et al. evaluated the antimicrobial activity of six irrigants on primary endodontic pathogens [32]. They found that MICs of doxycycline for all organisms tested (Peptostereptococcus micros, Prevotella intermedia, Stereptococcus sanguis and Lactobacillus acidophilus) were below 0.78μg/mL. LeCorn et al. studied the activity of several antibiotics against oral Actinomyces [33]. The MIC90 was 0.25μg/mL for doxycycline. They stated that the incorporation of these antimicrobial agents into the periradicular tissues through the root canal or as an intracanal irrigant may be effective in sterilizing lesions of endodontic origin, thus decreasing failure due to infection by Actinomy-ces. According to the results of the mentioned studies [28][29][30][31][32][33], it might be concluded that the RDXs in many of subgroups especially in DX5, DX10 and DX20 inhibit majority of pathogens that are involved in odontogenic infections.

In addition, fungi may be involved in cases of persistent and secondary periradicular lesions, the spectrum of antimicrobial activity of endodontic medicaments and irrigants should include these microorganisms. Himani et al. resulted that the doxycycline hydrochloride can be used effect-ively against Candida albicans [34].

In comparison of RDXs with MICs of doxycycline it should be considered that, according to many studies, MICs for planktonic microorganisms are not reliable predictors of the effects of doxycycline against their biofilms [35].

The inhibition of bone resorption by doxycycline occurs even when a low sub-antimicrobial dose is administered. Interestingly, chemically modified types of tetracyclines which lack any antibacterial effects are still effective in the inhibition of bone resorption [36].

The use of antibiotics as an intracanal medicament or irrigant may have potential side effects including crown discoloration, development of resistant bacterial strains, and allergic reaction [37]. Clinical and experimental studies have confirmed that different types of tetracycline, when use as intracanal medicaments (ledermix paste and triple antibiotic mixture) or irrigant (MTAD), cause varying degrees of discoloration [38][39][40]. Thus, incorporation of doxycycline to sealer might result in staining of teeth but such effects can be minimized if placement of the sealer is restricted to below the gingival margin.

Furthermore, the physicomechanical properties of endodontic sealers might be affected by adding antibiotics. Accordingly, Razmi et al. showed that adding amoxicillin or doxycycline at concentration of 1% to AH26 sealer led to a decrease in flow of the sealer, changes in setting time of AH26 i.e. decrease in setting time with amoxicillin and increase with doxycycline, increase in film thickness, increase in the dimensional changes following setting, and increase in the solubility of the sealers [10]. However, all changes were still within the BS EN ISO 6876 (2001) standards.

## CONCLUSION

In conclusion, under the conditions of this ex vivo study, doxycycline can be released from AH26 sealer-antibiotic combination through 3mm of apical root and apical foramen at 30 min, 48 and 72 h after mixing the sealer with doxycycline at concentrations of 0.5%, 1%, 2%, 5%, 10%, 20%. The AH26 sealer-antibiotic combination is not clinically approved to use in root canal treatment. This mixture could change the positive specification of AH26, such as biocompatibility, sealing ability, etc. Further studies are needed to evaluate the effect of antibiotic on biocompatibility as well as sealing properties of an endodontic sealer.
